# Application of Simultaneous Active and Passive Fluorescence Observations: Extending a Fluorescence-Based *q*_L_ Estimation Model

**DOI:** 10.3390/s25061700

**Published:** 2025-03-09

**Authors:** Chenhui Guo, Zhunqiao Liu, Xiaoliang Lu

**Affiliations:** 1 College of Natural Resources and Environment, Northwest A&F University, Yangling 712100, China; guochenhui@nwafu.edu.cn; 2 State Key Laboratory of Soil Erosion and Dryland Farming on the Loess Plateau, Northwest A&F University, Yangling 712100, China; zliu@nwafu.edu.cn

**Keywords:** broadband chlorophyll fluorescence spectra, fraction of open PSII reaction centers (*q*_L_), pulse-amplitude modulation (PAM), photosystem II (PSII), leaf level

## Abstract

The fraction of open Photosystem II (PSII) reaction centers (*q*_L_) is critical for connecting broadband PSII fluorescence (ChlF_PSII_) with the actual electron transport from PSII to Photosystem I. Accurately estimating *q*_L_ is fundamental for determining ChlF_PSII_, which, in turn, is vital for mechanistically estimating the actual electron transport rate and photosynthetic CO_2_ assimilation. Chlorophyll fluorescence provides direct physiological insights, offering a robust foundation for *q*_L_ estimation. However, uncertainties in the ChlF_PSII_–*q*_L_ relationship across different plant functional types (PFTs) limit its broader application at large spatial scales. To address this issue, we developed a leaf-level instrument capable of simultaneously measuring actively and passively induced chlorophyll fluorescence. Using this system, we measured light response, CO_2_ response, and temperature response curves across 52 species representing seven PFTs. Our findings reveal the following: (1) a strong linear correlation between ChlF_PSII_ derived from passively induced fluorescence and that from actively induced fluorescence (*R*^2^ = 0.85), and (2) while the parameters of the ChlF_PSII_–*q*_L_ relationship varied among PFTs, ChlF_PSII_ reliably modeled *q*_L_ within each PFT, with the *R*^2^ ranging from 0.85 to 0.96. This study establishes quantitative ChlF_PSII_–*q*_L_ relationships for various PFTs by utilizing passively induced fluorescence to calculate ChlF_PSII_. The results demonstrate the potential for remotely sensed chlorophyll fluorescence data to estimate *q*_L_ and strengthen the use of fluorescence-based approaches for mechanistic GPP estimation at large spatial scales.

## 1. Introduction

Light energy absorbed by plants is primarily dissipated through three pathways: driving electron transport, re-emission as chlorophyll fluorescence in the long-wavelength range (640–850 nm), or dissipation as heat [1,2]. Chlorophyll fluorescence is highly sensitive to variations in light intensity [3,4]. The temporal variation in chlorophyll fluorescence induced by actively applying additional light beyond ambient light conditions is referred to as chlorophyll fluorescence kinetics [5,6]. Numerous studies have leveraged chlorophyll fluorescence kinetics to rapidly assess photosynthetic physiological processes, leading to the development of various active fluorometers, such as pulse amplitude-modulated (PAM) fluorometers [7,8]. These advances have positioned chlorophyll fluorescence as a pivotal tool in photosynthesis research [9]. Recent advances in retrieving narrowband (e.g., the telluric oxygen absorption at 760 nm) solar-induced chlorophyll fluorescence (SIF) from various platforms (e.g., ground [10], air-borne [11], and satellite [12]) have created significant interest in its potential applications for estimating gross primary productivity (GPP) and analyzing photosynthetic physiological processes [13,14]. However, most current applications of SIF rely on empirical SIF-GPP relationships, which limits the universality and scalability of GPP estimation using SIF [15]. This limitation arises because the retrieved narrowband SIF provides limited physiological information, as it can only be retrieved under stable light conditions [13,16].

Gu et al. [17] introduced a mechanistic light reaction (MLR) model linking broadband chlorophyll fluorescence from Photosystem II (PSII) to the actual electron transport rate, drawing on classical chlorophyll fluorescence kinetics and SIF emission theory. Subsequent studies have shown that the MLR model has good potential to accurately estimate the photosynthetic carbon fixation at both the leaf and canopy scales [18,19,20]. However, it should be noted that the fraction of open PSII reaction centers (*q*_L_), a key parameter of the MLR model, is difficult to observe directly at scales beyond the leaf level. Currently, most *q*_L_ estimation methods rely on empirical relationships with PAR, which may fail to account for the influence of other environmental factors (e.g., temperature) and plant physiological dynamics on *q*_L_ [21,22]. Based on a global sensitivity analysis of a recently developed mechanical model of photosynthesis [23], Liu et al. [22] identified that the broadband chlorophyll fluorescence emitted from PSII (ChlF_PSII_) and leaf temperature (*T*_Leaf_) are the two major predictors of *q*_L_. Notably, in comparison with *q*_L_ estimations based on PAR, *q*_L_ derived from ChlF_PSII_ is better at capturing the diurnal dynamics of GPP [22]. The estimation of *q*_L_ at large scales using ChlF_PSII_ requires fitting the parameters of the ChlF_PSII_–*q*_L_ relationship for different species. Therefore, it is essential to develop instruments capable of concurrently measuring actively and passively induced chlorophyll fluorescence, thereby enabling the establishment of the ChlF_PSII_–*q*_L_ relationships across diverse species.

To date, many studies have focused on simultaneous observation of actively and passively induced chlorophyll fluorescence in relation to photosynthesis. For example, Magney et al. [24] modified the transparent chamber of a portable gas exchange system by integrating it with a PAM fluorometer and a spectrometer, enabling the simultaneous observation of gas exchange and chlorophyll fluorescence from the adaxial side of the leaf. Similarly, Viflan et al. [25] coupled a PAM instrument and a spectrometer with a portable gas exchange system to enable simultaneous measurements of gas exchange alongside PAM parameters (i.e., active chlorophyll fluorescence) from the adaxial side of the leaf and spectrally resolved chlorophyll fluorescence (i.e., passive chlorophyll fluorescence) from the abaxial side. Notably, both methods employ the transparent chamber of a photosynthesis system, external light sources as incident light, and an independently operated PAM fluorometer. However, the integration of multiple instruments may introduce potential uncertainties arising from variability among the instruments.

To minimize such errors, Meeker et al. [26] employed the Multiphase Flash^TM^ Fluorometer (LI-COR Inc., Lincoln, NE, USA), an integrated system combining a gas exchange measurement setup, PAM fluorometer, and incident light source, as a replacement for the transparent chamber. By modifying the bottom chamber plate of the Multiphase Flash^TM^ Fluorometer, they enabled the measurement of passively induced chlorophyll fluorescence spectra from the abaxial side of the leaf, in addition to conducting gas exchange and PAM parameter measurements. While these studies successfully achieved simultaneous observations of actively and passively induced chlorophyll fluorescence along with gas exchange, they were limited to chlorophyll fluorescence measurements from a single side of the leaf, which hinders the estimation of fluorescence at the photosystem scale [27,28].

In this study, we introduce a system capable of simultaneously measuring broadband chlorophyll fluorescence spectra from both sides of the leaf, along with actively induced chlorophyll fluorescence, and leaf-level gas exchange. The design, calibration, and measurement protocols of the system are detailed, followed by the construction of a chlorophyll fluorescence-based *q*_L_ estimation model using experimental data collected from multiple species representing diverse plant functional types (PFTs).

## 2. Materials and Methods

### 2.1. Instrument Design

**Figure 1:**
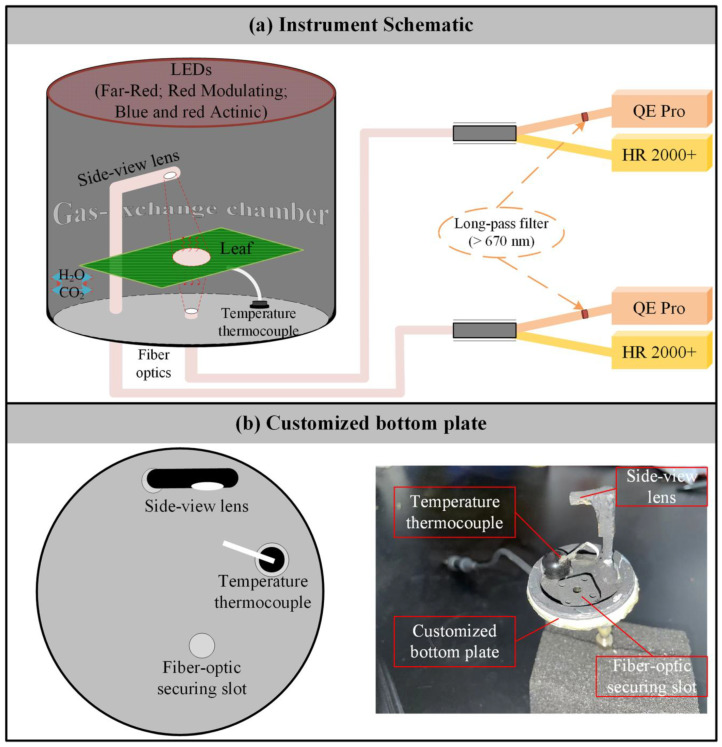
Overview of the developed system. (**a**) Schematic of the system setup. (**b**) Schematic and photograph of the custom metal plate.

We developed a leaf-level measurement system capable of concurrently measuring gas exchange, reflected and transmitted radiance, actively induced chlorophyll fluorescence, and the spectra of passively induced chlorophyll fluorescence from both sides of a leaf (Figure 1a). The system integrates a Multiphase Flash^TM^ Fluorometer from the LI-6800 portable photosynthesis system (LI-COR Inc., Lincoln, NE, USA) with two HR2000+ high-sensitivity spectrometers (Ocean Optics Inc., Dunedin, FL, USA) and two QE Pro high-sensitivity spectrometers (Ocean Optics Inc., Dunedin, FL, USA). The HR2000+ spectrometers cover a broad wavelength range of 296–1203 nm, providing an optical resolution of 5.316 nm and a spectral sampling interval of 0.443 nm. In contrast, the QE Pro spectrometers are optimized for a narrower range of 634–863 nm, offering higher optical resolution at 5.266 nm and a finer spectral sampling interval of 0.219 nm.

The bottom plate of the Multiphase Flash^TM^ Fluorometer was replaced with a custom-designed metal plate containing three airtight slots: one for securing the temperature sensor and two for holding bifurcated optical fibers of identical design (Figure 1b). One fiber was positioned vertically upward with a 25° field of view to collect downward emissions from the leaf. Its bifurcated ends were connected to an HR2000+ spectrometer for measuring transmitted radiation (*Rad*_DOWN_, mW m^−2^ nm^−1^ sr^−1^), and a QE Pro spectrometer for detecting downward chlorophyll fluorescence (ChlF_DOWN_, mW m^−2^ nm^−1^ sr^−1^). The second fiber was connected to a 30° field-of-view side-view lens oriented at a 45° zenith angle to observe upward spectral emissions from the leaf. This setup allowed the HR2000+ spectrometer to collect reflected radiation (*Rad*_UP_, mW m^−2^ nm^−1^ sr^−1^) and the QE Pro spectrometer to capture upward chlorophyll fluorescence from the adaxial side of the leaf (ChlF_UP_, mW m^−2^ nm^−1^ sr^−1^). Additionally, the distance between the side-view lens and the leaf was carefully adjusted to ensure alignment of the observation areas for both the upward- and downward-facing fibers (Figure 1b).

Notably, to reduce the impact of excitation light emitted by the Multiphase Flash^TM^ Fluorometer (blue light centered at 475 nm and red light centered at 625 nm) on chlorophyll fluorescence spectra, long-pass filters (670 nm) were inserted at the ports of each QE Pro spectrometer (Figure 1a). This configuration allows for the measurement of ChlF_DOWN_ and ChlF_UP_ within the wavelength range of 670–800 nm.

### 2.2. Calibrations

**Figure 2:**
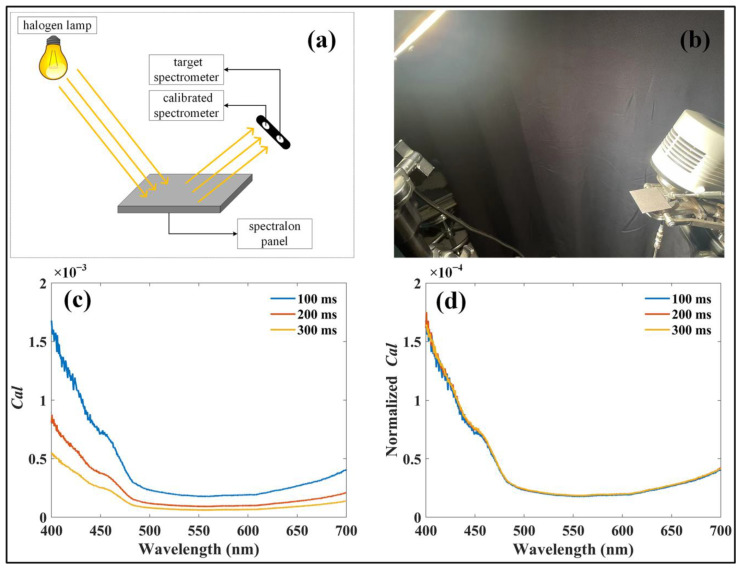
Calibration procedures for the developed system. (**a**) Schematic representation of the calibration process. (**b**) Laboratory setup during system calibration. (**c**) Calibration factors calculated for a spectrometer at different integration times. (**d**) Calibration factors normalized to integration time.

Before applying the system, radiometric calibration of the optical paths in the four spectrometers was performed using the cross-calibration method described by Yang et al. [29]. This method employs a pre-calibrated spectrometer as a reference to calibrate other spectrometers. In this study, a halogen lamp (Lowel Pro-light; Lowel-Light Manufacturing Inc., New York, NY, USA; 350–2500 nm, 250 W, 230/240 V AC~) was employed as the light source, and reflected radiance from a standard reflectance panel (Jingyi Optoelectronics Technology, Guangzhou, GD, China; ~2% reflectance) was measured simultaneously using a pre-calibrated spectrometer (QE Pro; Ocean Optics Inc., Dunedin, FL, USA) and the target spectrometers, which required calibration (Figure 2a,b). The calibration factor (*Cal*) was subsequently derived as follows:

(1) $$Cal=\frac{Rad}{{\mathrm{DN}}_{\mathrm{raw}}-{\mathrm{DN}}_{\mathrm{dark}}}$$

where *Rad* (mW m^−2^ nm^−1^ sr^−1^) represents the radiance of the light source, as measured by the pre-calibrated spectrometer; DN_raw_ designates the digital number recorded by each target spectrometer with the light source active; DN_dark_ represents the dark current recorded by each target spectrometer with the light source inactive.

Owing to the high signal-to-noise ratio of the spectrometers, the calculated *Cal* exhibited an approximately linear relationship with the integration time (Figure 2c,d). As a result, *Cal* normalized by the integration time was used to convert the recorded digital numbers into radiometric values (Figure 2d). It is crucial to highlight that, once the radiometric calibration is completed, the optical setup must remain unchanged, with the optical fiber firmly connected to the spectrometer until the experiment is completed.

### 2.3. Experiment Design

This section outlines the protocol for measuring response curves under varying environmental conditions (light, CO_2_, and temperature) using the developed system. Prior to the measurements, the target leaf was fully dark-adapted and positioned in the chamber under dark conditions, with its adaxial surface facing the light source, ensuring full coverage of the chamber. The initial chamber conditions were set as follows: light intensity of 0 μmol m^−2^ s^−1^, air flow rate of 500 μmol s^−1^, CO_2_ concentration of 420 μmol mol^−1^, leaf temperature of 25 °C, and relative humidity of 50%. Measurements of minimum (F_o_) and maximum fluorescence in the dark-adapted state (F_m_), along with dark respiration (*R*_d_, μmol m^−2^ s^−1^), were taken before increasing the light intensity. The light response curve was subsequently recorded at light intensities of 30, 50, 100, 200, 400, 600, 900, 1200, 1500, 1800, and 2100 μmol m^−2^ s^−1^. At each intensity, a stabilization period of 5–20 min was allowed for the gas exchange to reach a steady state. Following stabilization, measurements of net photosynthesis (*A*_net_, μmol m^−2^ s^−1^), steady-state fluorescence (F_s_), and minimum (F_o_′) and maximum fluorescence in the light-adapted state (F_m_′) were recorded, along with *Rad*_UP_, *Rad*_DOWN_, ChlF_UP_, and ChlF_DOWN_ corresponding to F_s_. The light intensity was then adjusted to the next level, and the procedure was repeated until the full light response curve was obtained. After completing the light response curve measurements, the CO_2_ response curve was recorded at a saturated light intensity of 1500 μmol m^−2^ s^−1^ using a series of CO_2_ concentrations: 420, 30, 50, 100, 200, 300, 400, 600, 900, 1200, and 1500 μmol mol^−1^. A stabilization period of 5–20 min was allowed at each concentration before recording the data, following the same protocol as for the light response curve. The temperature response curve was then measured while maintaining a constant light intensity of 1500 μmol m^−2^ s^−1^ and a CO_2_ concentration of 420 μmol mol^−1^. The leaf temperature was sequentially adjusted to 25, 15, 20, 25, 30, 35, 40, and 45 °C. In the same way as for the other response curves, the gas exchange was allowed to stabilize for 5–20 min before the data collection for each temperature. Note that throughout all the measurements, the leaf remained fixed in its position within the chamber to ensure consistency.

After completing the environmental response curve measurements, reflectance and transmittance spectra (hereafter referred to as *ρ* and *τ*) across the wavelength range of 350–2500 nm were measured on the same leaf using a Fluowat leaf clip and a spectrometer (PSR+ 3500; Spectral Evolution, MA, USA; 1 nm spectral resolution, 350–2500 nm). The radiation characteristics of the light source were also evaluated using a standard reflectance panel (Guangzhou Jingyi Photoelectric Technology Co., Ltd., Guangzhou, China; ~2% reflectance).

In this study, we conducted concurrent measurements of leaf gas exchange and actively and passively induced chlorophyll fluorescence across 52 species representing major plant functional types (Table S1). All plant samples were obtained from the Museum Garden, Northwest A&F University (https://bly.nwsuaf.edu.cn/English/index.htm (accessed on 1 July 2022)), which is situated in a sub-humid continental monsoon climate zone with a mean annual precipitation of 630 mm and an average annual temperature of 12.9 °C [30]. Due to the difficulty of performing dark adaptation in the field, the experiment was carried out in a controlled environment at the State Key Laboratory of Soil Erosion and Dryland Farming on the Loess Plateau, Northwest A&F University, Yangling, China (108°04′ E, 34°17′ N), from July to August 2022. To prevent xylem embolism, branches were excised underwater in the morning, following best practices [31], and then dark-adapted for one hour prior to the measurements. Healthy, fully expanded leaves from the prepared branches were then selected for measurements using the methods described above.

### 2.4. Estimation of q_L_

According to Liu et al. [22], *q*_L_ can be modeled as follows:

(2) $$q_{L\_\mathrm{MOD}}=\frac{m}{{{\mathrm{ChlF}}_{\mathrm{PSII}}}^{1/m}+m}$$

where *q*_L_MOD_ represents the *q*_L_ simulated from ChlF_PSII_; ChlF_PSII_ (μmol m^−2^ s^−1^) indicates the broadband chlorophyll fluorescence from PSII; *m* is a dimensionless parameter representing the impact of temperature on *q*_L_ simulation. Liu et al. [22] demonstrated that *m* can be modeled by a peaked function:

(3) $$m=m_{\mathrm{opt}}\times \frac{H_{d}\times \exp\left(\frac{H_{a}\times \left(T_{\mathrm{leaf}\_K}-T_{\mathrm{opt}}\right)}{T_{\mathrm{leaf}\_K}\times R\times T_{\mathrm{opt}}}\right)}{H_{d}-H_{a}\times \left[1-\exp\left(\frac{H_{d}\times \left(T_{\mathrm{leaf}\_K}-T_{\mathrm{opt}}\right)}{T_{\mathrm{leaf}\_K}\times R\times T_{\mathrm{opt}}}\right)\right]}$$

where *m*_opt_ represents the *m* at the optimal leaf temperature; *T*_leaf_K_ is the leaf temperature in Kelvin (K); *T*_opt_ is the optimal leaf temperature (K); *H*_d_ and *H*_a_ are the rates of decline and increase in the peaked function above and below the optimal leaf temperature (J mol^−1^), respectively; *R* is the universal gas constant (8.314 J mol^−1^ K^−1^).

Here, we utilized the observed data to determine the parameters required for estimating *q*_L_, namely *m*_opt_, *H*_d_, *H*_a_, and *T*_opt_. Using Equation (2), parameter *m* was fitted using *q*_L_ and ChlF_PSII_, both derived from measurements across varying temperatures with the developed system. Subsequently, the fitted values of *m* and their corresponding temperatures were used to estimate the parameters in Equation (3), i.e., *m*_opt_, *H*_d_, *H*_a_, and *T*_opt_. Below, we describe the methods for obtaining *q*_L_ and ChlF_PSII_ from the collected measurements.

Based on the lake model, *q*_L_ can be derived from PAM parameters (*q*_L_PAM_) [32]. Specifically,

(4) $$q_{L\_\mathrm{PAM}}=\frac{F_{m}\prime -F_{s}}{F_{m}\prime -F_{o}\prime }\times \frac{F_{o}\prime }{F_{s}}$$

In this study, ChlF_PSII_ was derived from passive chlorophyll fluorescence (ChlF_PSII_fPSII_) following the method of Liu et al. [19]:

(5) $${\mathrm{ChlF}}_{\mathrm{PSII}\_\mathrm{fPSII}}={\mathrm{ChlF}}_{\mathrm{PS}\_\lambda }\times f_{\mathrm{PSII}\_\lambda }\times f_{C\_\lambda }$$

where ChlF_PS_λ_ (mW m^−2^ nm^−1^) is the total chlorophyll fluorescence at the photosystem level at λ nm; *f*_PSII_λ_ represents the contribution of PSII to the total chlorophyll fluorescence at λ nm; *f*_C_λ_, derived from the elementary fluorescence emission spectrum of PSII (Figure S1), represents the conversion factor translating the photosystem-level PSII chlorophyll fluorescence at λ nm (in units of mW m^−2^ nm^−1^) into ChlF_PSII_FULL_; λ ranges from 640 to 850 nm.

ChlF_PS_λ_ can be derived by correcting leaf-emitted chlorophyll fluorescence spectra (i.e., ChlF_UP_ and ChlF_DOWN_) [27]:

(6) $${\mathrm{ChlF}}_{\mathrm{PS}\_\lambda }=\frac{{\mathrm{ChlF}}_{\mathrm{UP}\_\lambda }+{\mathrm{ChlF}}_{\mathrm{DOWN}\_\lambda }}{\rho_{\_\lambda }+\tau_{\_\lambda }}\times \pi$$

SIF retrieval on ground-based platforms often selects absorption bands with wide and deep absorption features, such as the O_2_-A band (~760 nm) [13]. Therefore, *f*_PSII_ at 760 nm (*f*_PSII_760_) was calculated following the method proposed by Guo et al. [33] to estimate ChlF_PSII_fPSII_ (see Supplementary Materials (Text S1) for details). It should be noted that this study also estimated *f*_PSII_ at 740 nm (*f*_PSII_740_), as satellite measurements typically target solar Fraunhofer lines that are less affected by atmospheric absorption, such as the band near 740 nm [34].

Here, we have validated the method for estimating ChlF_PSII_ from passive chlorophyll fluorescence by comparing ChlF_PSII_fPSII_ with ChlF_PSII_ derived from PAM parameters (ChlF_PSII_PAM_) following the method of Han et al. [18]

## 3. Results

### 3.1. Light Response Curve Measurements

**Figure 3:**
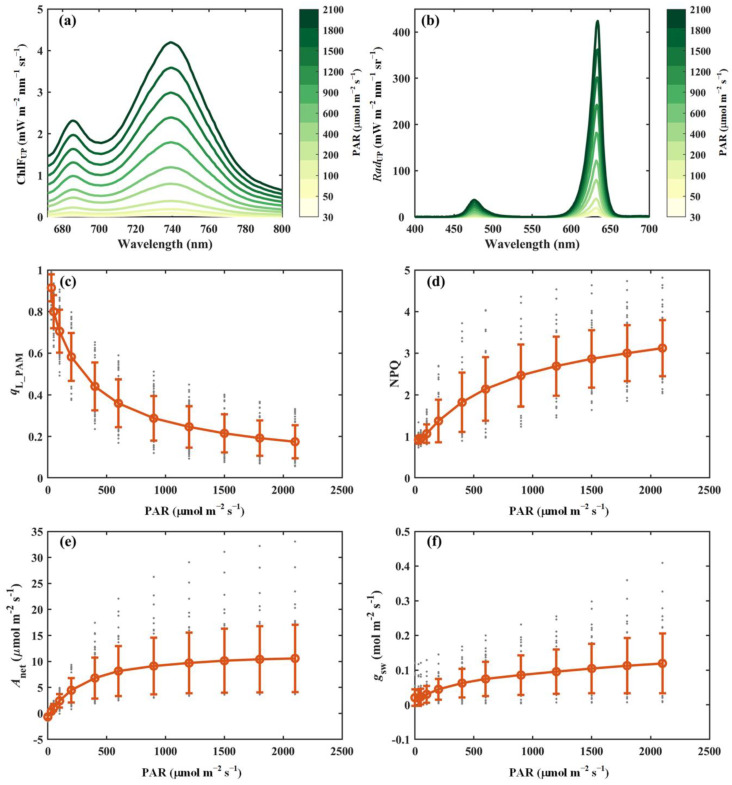
Representative results from light response curve measurements. (**a**) Upward chlorophyll fluorescence (ChlF_UP_, mW m^−2^ nm^−1^ sr^−1^). (**b**) Reflected radiance (*Rad*_UP_, mW m^−2^ nm^−1^ sr^−1^). (**c**) Fraction of open PSII reaction centers derived from PAM parameters (*q*_L_PAM_). (**d**) Non-photochemical quenching derived from PAM parameters (NPQ). (**e**) Net photosynthesis (*A*_net_, μmol m^−2^ s^−1^). (**f**) Stomatal conductance (*g*_sw_, mol m^−2^ s^−1^). In panels (**c**–**f**), the red circles and error bars represent the mean values and corresponding standard deviations of 52 species, respectively, while the gray dots indicate the measurements for individual species.

This section presents representative results from the light response curve measurements obtained using the developed system (Figure 3). The upward chlorophyll fluorescence (i.e., ChlF_UP_) exhibited an asymmetric spectral distribution, characterized by lower emission intensity in the red region (<700 nm) and higher intensity in the near-infrared region (>700 nm), primarily due to reabsorption within the leaf tissues (Figure 3a). Although the overall spectral shape of ChlF_UP_ remained largely consistent across varying light intensities, its absolute values varied substantially. Specifically, the red chlorophyll fluorescence emission peak (around 686 nm) increased from 0.04 mW m^−2^ nm^−1^ sr^−1^ at a light intensity of 30 μmol m^−2^ s^−1^ to about 2.3 mW m^−2^ nm^−1^ sr^−1^ at 2100 μmol m^−2^ s^−1^, whereas the near-infrared fluorescence peak (around 740 nm) rose from 0.05 mW m^−2^ nm^−1^ sr^−1^ to 4.2 mW m^−2^ nm^−1^ sr^−1^ over the same range (Figure 3a). Simultaneously, leaf-reflected radiation (i.e., *Rad*_UP_) was captured (Figure 3b). Notably, the ratio of *Rad*_UP_ in the red wavelength to that in the blue wavelength varied with light intensity, potentially influenced by chloroplast movement, which may alter light absorption characteristics. The combined measurements of *Rad*_UP_ and *Rad*_DOWN_ provide valuable insights into accurately estimating the fraction of absorbed energy.

In addition to passively induced chlorophyll fluorescence, actively induced chlorophyll fluorescence responded as expected to increasing light intensity. For example, as the light intensity increased from 30 to 2100 μmol m^−2^ s^−1^, *q*_L_PAM_ declined progressively from 0.91 to approximately 0.17. In contrast, non-photochemical quenching (NPQ) exhibited an upward trend, rising from 0.92 to a stable value of about 3.12 under high light conditions. The responses of *A*_net_ and stomatal conductance (*g*_sw_) to light intensity are also presented in Figure 3e,f. Both *A*_net_ and *g*_sw_ increased rapidly at low light levels, eventually reaching saturation at higher intensities. At saturation light intensity, *A*_net_ and *g*_sw_ stabilized at approximately 10 μmol m^−2^ s^−1^ and 0.11 mol m^−2^ s^−1^, respectively.

### 3.2. Estimation of f_PSII_760_ and ChlF_PSII_fPSII_

**Figure 4:**
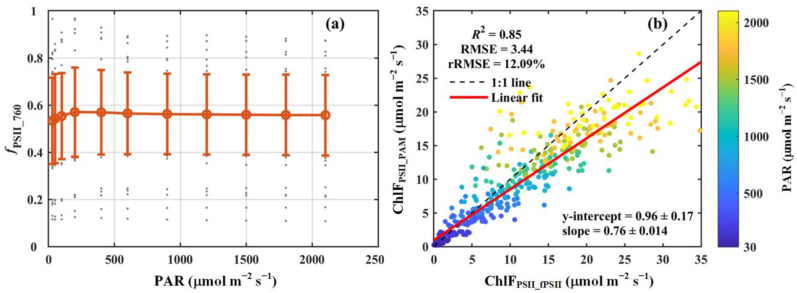
(**a**) The light response curve of the contribution of PSII to total chlorophyll fluorescence at 760 nm (*f*_PSII_760_). (**b**) Comparison of the broadband chlorophyll fluorescence from PSII estimated using *f*_PSII_760_ (ChlF_PSII_fPSII_) with that derived from PAM measurements (ChlF_PSII_PAM_). The red circles and error bars in panel (**a**) denote the means and standard deviations for 52 species, respectively, while the gray dots show the results for each species. In panel (**b**), the black dashed line and red solid line indicate the 1:1 line and the linear regression result, respectively. The color scheme indicates photosynthetically active radiation (PAR, μmol m^−2^ s^−1^). The coefficient of determination (*R*^2^), root-mean-square error (RMSE, μmol m^−2^ s^−1^), and relative root-mean-square error (rRMSE, %) are reported. The regression slope and intercept are also included.

We observed that under low light conditions (<500 μmol m^−2^ s^−1^), *f*_PSII_760_ exhibited a pronounced response to changes in light intensity, increasing from 0.53 to 0.58 as the light intensity rose from 30 to 500 μmol m^−2^ s^−1^. At higher light intensities (>500 μmol m^−2^ s^−1^), *f*_PSII_760_ displayed minimal variation, stabilizing at approximately 0.56 (Figure 4a). While *f*_PSII_760_ showed moderate variability across light intensities, notable differences were observed among the seven PFTs analyzed in this study (Table 1). Specifically, deciduous needleleaf forest (DNF) had the lowest mean *f*_PSII_760_ value (about 0.27), whereas evergreen broadleaf forest (EBF) had the highest (about 0.71), which was 2.5 times greater than that of DNF. In contrast to the substantial differences observed among the PFTs, variability within individual PFTs was relatively small, except for DNF. Within each PFT, the standard deviations of *f*_PSII_760_ were substantially lower than their respective means, indicating relatively consistent values within the PFTs (Table 1).

In addition, our results reveal a strong correlation between ChlF_PSII_fPSII_ and ChlF_PSII_PAM_. Across all species, ChlF_PSII_fPSII_ accounted for approximately 85% of the variance in ChlF_PSII_PAM_, with an RMSE of 3.44 μmol m^−2^ s^−1^ and an rRMSE of 12.09% (Figure 4b). A slight overestimation in ChlF_PSII_fPSII_ (slope = 0.76) was observed, likely attributable to the omission of PSI contributions to the total chlorophyll fluorescence at 686 nm during the calculation of *f*_PSII_760_. For the different PFTs, ChlF_PSII_fPSII_ also explained the majority of the variation in ChlF_PSII_PAM_, with *R*^2^ values ranging from 0.79 to 0.99. The RMSE varied between 0.58 and 4.66 μmol m^−2^ s^−1^, while the rRMSE remained below 20%, ranging from 2.03% to 18.96% (Table 1).

### 3.3. q_L_ Estimation Results

**Figure 5:**
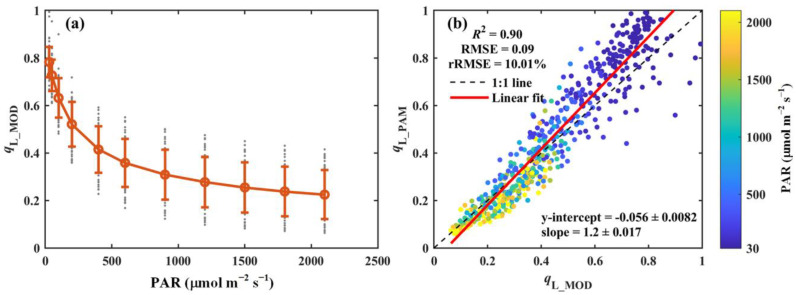
(**a**) Light response curve of the modeled *q*_L_ (*q*_L_MOD_). (**b**) Comparison of the modeled *q*_L_ (*q*_L_MOD_) with that derived from PAM measurements (*q*_L_PAM_). The red circles and error bars in panel (**a**) denote the means and standard deviations for 52 species, respectively, while the gray dots show the results for each species. In panel (**b**), the black dashed line and red solid line indicate the 1:1 line and the linear regression result, respectively. The color scheme indicates photosynthetically active radiation (PAR, μmol m^−2^ s^−1^). The coefficient of determination (*R*^2^), root-mean-square error (RMSE), and relative root-mean-square error (rRMSE, %) are reported. The regression slope and intercept are also included.

The modeled *q*_L_ (*q*_L_MOD_), using the method proposed by Liu et al. [22], is presented in Figure 5. Similarly to *q*_L_PAM_, *q*_L_MOD_ exhibits a strong response to variations in light intensity, decreasing sharply under low light conditions and stabilizing as light intensity increases (Figure 5a). A modest underestimation of *q*_L_MOD_ was observed under low light conditions, possibly due to the absence of low-light measurements in the dataset used to fit the empirical parameters. Despite this underestimation, *q*_L_MOD_ accounted for 90% of the variation in *q*_L_PAM_, with an RMSE and rRMSE of 0.09 and 10.01%, respectively (Figure 5b). These results suggest that the fitted empirical parameters (Table S1) provide a robust framework for modeling *q*_L_ across a wide range of environmental conditions.

The proposed model demonstrated a strong performance in estimating *q*_L_ across all seven PFTs. *q*_L_MOD_ closely aligns with *q*_L_PAM_ for all PFTs, with *R*^2^ values exceeding 0.85 and rRMSE values below 12.66% (Table 2). Notably, the model performed best in cropland (CRO) (*R*^2^ = 0.96, RMSE = 0.1, and rRMSE = 11.24%, Table 2). While the weakest correlation was observed in shrubland (SHR), *q*_L_MOD_ still explained 85% of the variation in *q*_L_PAM_, with RMSE and rRMSE values of 0.11 and 12.66%, respectively (Table 2).

## 4. Discussion

*q*_L_ plays a pivotal role in linking PSII chlorophyll fluorescence emission to the actual electron transport rate from PSII to PSI [32]. The accurate estimation of *q*_L_ facilitates a quantitative understanding of the relationship between ChlF_PSII_ and the electron transport rate required for carbon metabolism, thereby providing a mechanistic foundation for leveraging chlorophyll fluorescence to estimate GPP [17]. In this study, we developed an advanced system capable of simultaneously measuring active and passive chlorophyll fluorescence alongside gas exchange, offering robust support for the precise quantification of *q*_L_.

Using data collected under varying temperature conditions (i.e., temperature response curves), we fitted the key parameters, namely *m*_opt_, *H*_d_, *H*_a_, and *T*_opt_, of the ChlF_PSII_–*q*_L_ relationship for 52 species across seven major PFTs (Table S1). With the fitted parameters, *q*_L_MOD_ exhibited strong agreement with *q*_L_PAM_ under various light conditions (Figure 5). In addition, we found that *f*_PSII_760_ exhibited minimal sensitivity to environmental variations, remaining stable under most conditions. This observation is consistent with results from a process-based model [20], reinforcing confidence in the reliability of *f*_PSII_760_ estimates. Furthermore, ChlF_PSII_ estimated using *f*_PSII_760_ closely matched ChlF_PSII_ derived from PAM measurements (Figure 4), highlighting the consistency between active and passive chlorophyll fluorescence measurements obtained with the developed system. We also calculated the PSII contribution at 740 nm (*f*_PSII_740_), as most satellite platforms retrieve SIF at this wavelength. The results show that *f*_PSII_740_ was higher than *f*_PSII_760_ due to the PSII fluorescence emission peak occurring around 740 nm. However, the PSII contribution exhibited a similar response to changes in light intensity at both 740 nm and 760 nm (Figure S2), indicating that near-infrared fluorescence at these wavelengths provides comparable physiological insights. The observed differences in the PSII contributions across the wavelengths in this region likely stem from the intrinsic properties of plant fluorescence emission rather than variations in plant physiological conditions. Therefore, selecting either 740 nm or 760 nm produces consistent results and does not influence the overall conclusions of this study. These findings provide a robust methodological foundation for estimating the broadband total SIF emitted from PSII and *q*_L_ using SIF data from remote sensing platforms.

It is important to note that the light response curves in this study were measured under a protocol in which light intensities transitioned from low to high, rather than the conventional protocol in which light intensities are adjusted from high to low [18]. This modification was implemented to address specific challenges associated with PAM parameters. Specifically, non-photochemical energy dissipation relaxes slowly during the transition from high to low light [35], resulting in deviations in PAM parameters collected at lower light levels, such as abnormally low values of the photochemical yield of PSII. By adopting a low-to-high light intensity sequence, we aimed to improve the reliability of PAM observational data. Although this protocol increased the measurement time for light response curves due to repeated photosynthetic light induction at each intensity, it significantly enhanced the quality of PAM parameters while maintaining the quality of other measurements, such as *A*_net_ and ChlF_UP_ (Figure 3). Furthermore, the low-to-high light intensity sequence offered additional advantages for subsequent environmental response curve measurements (i.e., CO_2_ and temperature response curves), which require saturating light conditions. By concluding light response curve measurements under high light, this approach eliminated the need for re-induction, streamlining the transition between measurements.

In addition to *q*_L_ modeling, the developed system offers significant potential for advancing the application of chlorophyll fluorescence in estimating carbon and water fluxes, as well as monitoring plant stress. Photosynthesis models following the Farquhar–von Caemmerer–Berry (FvCB) framework [36] rely on the maximum carboxylation rate (*V*_cmax_) to simulate gas exchange fluxes associated with photosynthesis, serving as essential tools for evaluating ecosystem carbon and water dynamics. A major challenge in scaling these models to broader spatial domains is the accurate estimation of the key parameters (e.g., *V*_cmax_), which remains a focal point of research [37]. Recent studies have highlighted the potential of SIF for estimating *V*_cmax_ [38,39,40]. The system developed here provides critical experimental data to support efforts aimed at utilizing chlorophyll fluorescence for such estimations. Moreover, chlorophyll fluorescence, predominantly driven by light intensity during the light reactions, occurs independently of the dark reactions that jointly govern photosynthesis [1]. Interestingly, our findings reveal that chlorophyll fluorescence can also reflect changes in dark reaction processes, such as variations in stomatal conductance induced by changes in CO_2_ concentration (Figure S3). These results underscore the system’s capacity to enhance plant stress research through chlorophyll fluorescence-based methods [41,42].

## 5. Conclusions

In this study, we developed an advanced observation system capable of simultaneously measuring gas exchange, actively induced chlorophyll fluorescence, and passively induced chlorophyll fluorescence. Using this system, we conducted comprehensive measurements of light response curves, CO_2_ response curves, and temperature response curves across 52 species representing seven PFTs. Our findings reveal that *f*_PSII_760_ exhibited limited sensitivity to environmental variations. Moreover, ChlF_PSII_ derived from *f*_PSII_760_ showed a strong correlation with that calculated based on PAM parameters (*R*^2^ = 0.85, RMSE = 3.44 μmol m^−2^ s^−1^, and rRMSE = 12.09%). Additionally, we extended the semi-empirical model for *q*_L_ estimation proposed by Liu et al. [22]. Our results demonstrate that the modeled *q*_L_MOD_ could explain 90% of the observed *q*_L_PAM_ variability (RMSE = 0.09 and rRMSE = 10.01%). With the increasing availability of satellite-based SIF observations, our findings provide a promising pathway for large-scale estimation of ChlF_PSII_ and *q*_L_. This advancement contributes to improving the ability to estimate GPP using SIF in a mechanistic framework, while also deepening our understanding of photosynthetic processes.

## Figures and Tables

**Table 1:** *f*_PSII_760_ values for different plant functional types (PFTs) and comparisons of ChlF_PSII_fPSII_ and ChlF_PSII_PAM_ for each PFT. *R*^2^, RMSE, and rRMSE are the coefficient of determination, root-mean-square error (μmol m^−2^ s^−1^), and relative root-mean-square error (%), respectively. ENF: evergreen needleleaf forest; EBF: evergreen broadleaf forest; DNF: deciduous needleleaf forest; DBF: deciduous broadleaf forest; SHR: shrubland; GRA: grass; CRO: cropland.

	ENF	EBF	DNF	DBF	SHR	GRA	CRO
*f*_PSII_760_	0.45 ± 0.15	0.71 ± 0.16	0.27 ± 0.18	0.52 ± 0.15	0.53 ± 0.15	0.64 ± 0.11	0.67 ± 0.14
*R* ^2^	0.85	0.79	0.99	0.84	0.86	0.92	0.95
RMSE	2.73	4.66	0.58	4.49	2.55	2.99	2.33
rRMSE	11.64	18.96	2.03	18.39	12.28	12.64	16.51

**Table 2:** Comparison of the model (*q*_L_MOD_) and measured *q*_L_ (*q*_L_PAM_) across different plant functional types. *R*^2^, RMSE, and rRMSE are the coefficient of determination, root-mean-square error, and relative root-mean-square error (%), respectively. ENF: evergreen needleleaf forest; EBF: evergreen broadleaf forest; DNF: deciduous needleleaf forest; DBF: deciduous broadleaf forest; SHR: shrubland; GRA: grass; CRO: cropland.

	ENF	EBF	DNF	DBF	SHR	GRA	CRO
*R* ^2^	0.93	0.92	0.86	0.90	0.85	0.95	0.96
RMSE	0.08	0.1	0.1	0.09	0.11	0.09	0.1
rRMSE	8.89	10.71	12.60	10.32	12.66	9.40	11.24

## Data Availability

The data used for the analyses are available at https://github.com/luxiaoliangnwafu/Dataset-multiSpecies (accessed on 8 March 2025).

## Supplementary Materials

The following supporting information can be downloaded at https://www.mdpi.com/article/10.3390/s25061700/s1: Text S1: Estimation of *f*_PSII_760_; Figure S1: Elementary fluorescence emission spectrum of Photosystem II (*S*_PSII_, unitless) obtained from the SCOPE model, Version 1.73; Figure S2. The light response curve of the contribution of PSII to total chlorophyll fluorescence at 760 nm (*f*_PSII_760_) and 740 nm (*f*_PSII_740_); Figure S3: CO_2_ response curve of (a) upward chlorophyll fluorescence (ChlF_UP_, mW m^−2^ nm^−1^ sr^−1^) and (b) downward chlorophyll fluorescence (ChlF_DOWN_, mW m^−2^ nm^−1^ sr^−1^). Temperature response curve of (c) upward chlorophyll fluorescence (ChlF_UP_, mW m^−2^ nm^−1^ sr^−1^) and (b) downward chlorophyll fluorescence (ChlF_DOWN_, mW m^−2^ nm^−1^ sr^−1^); Table S1: The fitted parameters (*m*_opt_, *H*_d_, *H*_a_, and *T*_opt_) of the ChlF_PSII_–*q*_L_ relationship for different species. References [43,44,45,46] are cited in the supplementary materials.

## Abbreviations

**Symbols**	**Definition**	**Unit**
*A*_net_	Net photosynthesis	μmol m^−2^ s^−1^
*g*_sw_	Stomatal conductance	mol m^−2^ s^−1^
*R*_d_	Dark respiration	μmol m^−2^ s^−1^
ChlF_DOWN_	Downward chlorophyll fluorescence	mW m^−2^ nm^−1^ sr^−1^
ChlF_UP_	Upward chlorophyll fluorescence	mW m^−2^ nm^−1^ sr^−1^
ChlF_PS_λ_	Chlorophyll fluorescence at the photosystem level at λ nm	mW m^−2^ nm^−1^
ChlF_PSII_	Broadband chlorophyll fluorescence emitted from PSII	μmol m^−2^ s^−1^
ChlF_PSII_fPSII_	ChlF_PSII_ derived from passive chlorophyll fluorescence	μmol m^−2^ s^−1^
ChlF_PSII_PAM_	ChlF_PSII_ derived from PAM parameters	μmol m^−2^ s^−1^
*f*_C_λ_	Conversion factor linking photosystem-level PSII chlorophyll fluorescence at λ nm to ChlF_PSII_	μmol nm mW^−1^ s^−1^
F_m_	Maximum fluorescence in the dark-adapted state	\
F_m_′	Maximum fluorescence in the light-adapted state	\
F_o_	Minimum fluorescence in the dark-adapted state	\
F_o_′	Minimum fluorescence in the light-adapted state	\
F_s_	Steady-state fluorescence in the light-adapted state	\
*f*_PSII_λ_	Contribution of PSII to chlorophyll fluorescence at λ nm	\
GPP	Gross primary productivity	μmol m^−2^ s^−1^
*m*	Fitted parameter for estimating *q*_L_MOD_	\
*m*_opt_	Value of m at optimal leaf temperature	\
*H*_a_	Fitted parameter for estimating m	J mol^−1^
*H*_d_	Fitted parameter for estimating m	J mol^−1^
*T*_opt_	Optimal leaf temperature for *q*_L_	K
*R*	Universal gas constant	J mol^−1^ K^−1^
NPQ	Non-photochemical quenching	\
*q*_L_	Fraction of open PSII reaction centers	\
*q*_L_MOD_	*q*_L_ estimated from ChlF_PSII_	\
*q*_L_PAM_	*q*_L_ derived from PAM	\
*Rad*_DOWN_	Transmitted radiation	mW m^−2^ nm^−1^ sr^−1^
*Rad*_UP_	Reflected radiation	mW m^−2^ nm^−1^ sr^−1^
SIF	Solar-induced chlorophyll fluorescence	mW m^−2^ nm^−1^ sr^−1^
*T*_leaf_	Leaf temperature	°C
*T*_leaf_K_	Leaf temperature in Kelvin	K
*V*_cmax_	Maximum carboxylation rate	μmol m^−2^ s^−1^
*Cal*	Calibration factor	mW m^−2^ nm^−1^ sr^−1^
*Rad*	Radiance of light source	mW m^−2^ nm^−1^ sr^−1^
DN_raw_	Digital number recorded by spectrometer	\
DN_dark_	Dark current recorded by spectrometer	\
*ρ*	Leaf reflectance	\
*τ*	Leaf transmittance	\
