# Supplementary material for: Application of Simultaneous Active and Passive Fluorescence Observations: Extending a Fluorescence-Based qL Estimation Model
Source: Sensors (Basel). 2025 Mar 9;25(6):1700. doi: 10.3390/s25061700 (PMC11946858; doi:10.3390/s25061700)
Supplement: Supplementary file 1 [file sensors-25-01700-s001.zip › sensors-3447345-supplementary.pdf]

Text S1. Estimation of f_{PSII_760}

At a specific wavelength, f_{PSII_λ} can be expressed as [1]:

$$f_{PSII\_\lambda} = \frac{ChlF_{PSII\_\lambda}}{ChlF_{PS\_\lambda}} \quad \text{(S1)}$$

where $ChlF_{PS\_\lambda}$ (mW m^{-2} nm^{-1}) is the total chlorophyll fluorescence at the photosystem level at λ nm; $ChlF_{PSII\_\lambda}$ (mW m^{-2} nm^{-1}) is the chlorophyll fluorescence emitted from PSII at the photosystem level at a given wavelength λ nm.

Given the stability of the spectral shape of PSII chlorophyll fluorescence at the photosystem level, theoretically determined by its elementary fluorescence emission spectrum (Figure S1), quantitative relationships can be established between different spectral bands of PSII chlorophyll fluorescence at the photosystem level:

$$\frac{ChlF_{PSII\_\lambda}}{ChlF_{PSII\_\lambda'}} = \frac{S_{PSII\_\lambda}}{S_{PSII\_\lambda'}} \quad \text{(S2)}$$

where λ and λ′ range from 640 to 850 nm; S_{PSII_λ} and $S_{PSII\_\lambda'}$ represent the values of the elementary fluorescence emission spectrum of PSII at λ and λ′ nm, respectively. Accordingly, PSII chlorophyll fluorescence at 760 nm can be derived from that at 686 nm:

$$ChlF_{PSII\_760} = ChlF_{PSII\_686} \times \frac{S_{PSII\_760}}{S_{PSII\_686}} \quad \text{(S3)}$$

where $ChlF_{PSII\_686}$ (mW m^{-2} nm^{-1}) and $ChlF_{PSII\_760}$ (mW m^{-2} nm^{-}) represent chlorophyll fluorescence emitted by PSII at the photosystem level at 686 and 760 nm, respectively.

Since the contribution of PSI in the red region is almost negligible relative to PSII chlorophyll fluorescence [2], PSII chlorophyll fluorescence in this spectral range can be approximated as the total chlorophyll fluorescence [3]. Thus, we have:

$$ChlF_{PSII\_686} \approx ChlF_{PS\_686} \tag{S4}$$

where $ChlF_{PS\_686}$ (mW m^{-2} nm^{-1}) represents the photosystem-level chlorophyll fluorescence at 686 nm, containing the contributions from both PSI and PSII.

By substituting Eqs. (S3) and (S4) into Eq. (S1), f_{PSII_760} can be estimated as:

$$f_{PSII\_760} = \frac{ChlF_{PS\_686}}{ChlF_{PS\_760}} \times \frac{S_{PSII\_760}}{S_{PSII\_686}} \tag{S5}$$

where $ChlF_{PS\_760}$ (mW m^{-2} nm^{-1}) represents the photosystem-level chlorophyll fluorescence at 760 nm. Based on the elementary fluorescence emission spectrum of PSII provided by the SCOPE model, Version 1.73, the ratio of S_{PSII_760} to S_{PSII_686} can be set to 0.086 [4, 5]. Consequently, f_{PSII_760} can be estimated as a function of $ChlF_{PS\_686}$ and $ChlF_{PS\_760}$.

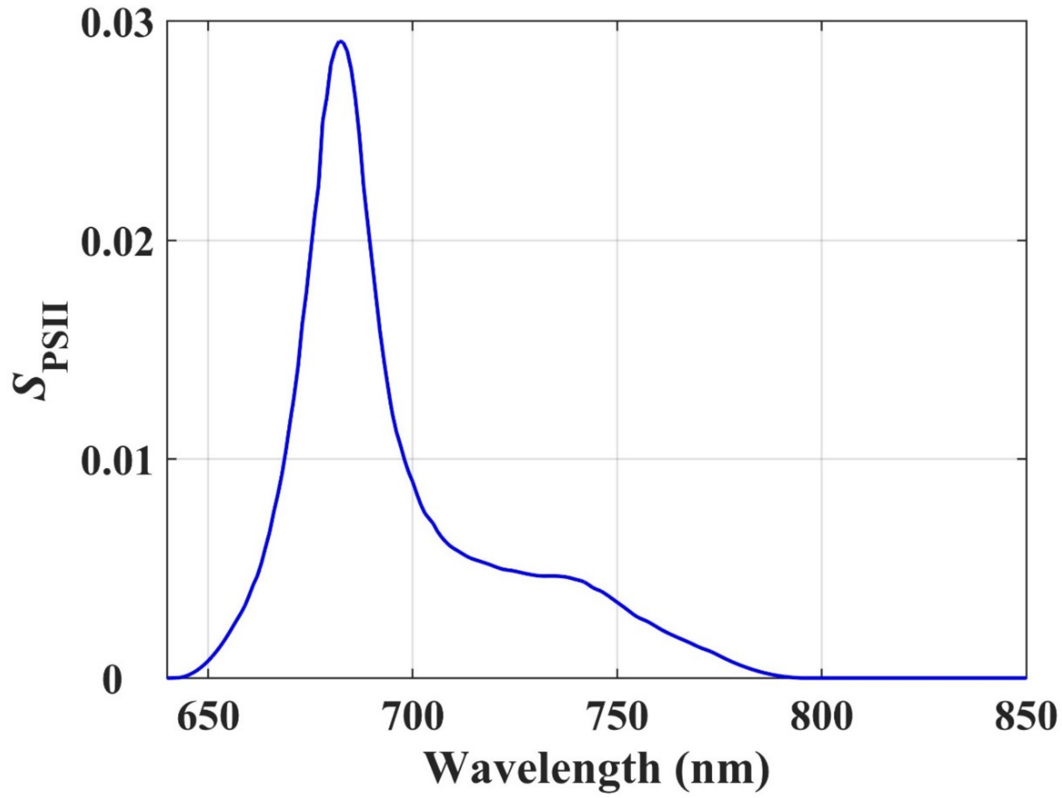

Figure S1. Elementary fluorescence emission spectrum of Photosystem II (S_{PSII}, unitless) obtained from the SCOPE model, Version 1.73 [4, 5].

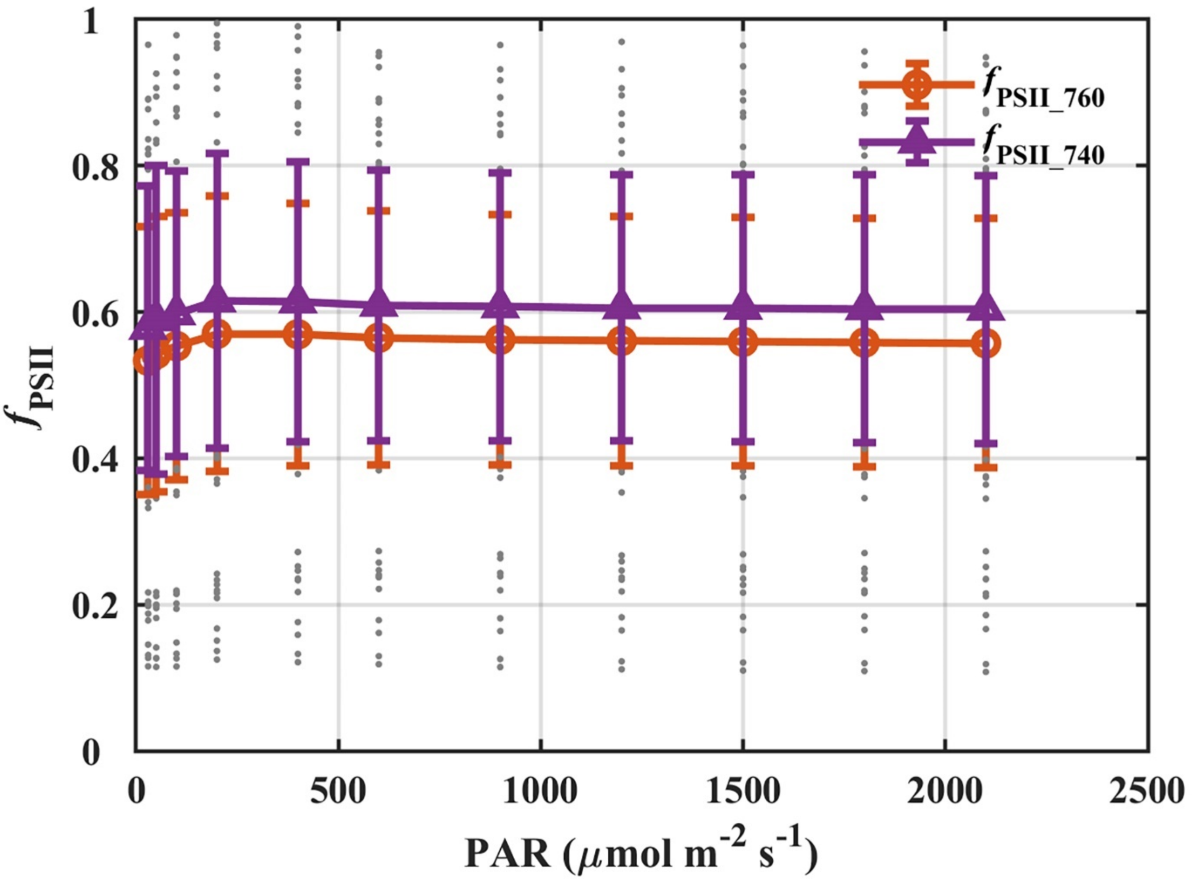

Figure S2. The light response curve of the contribution of PSII to total chlorophyll fluorescence at 760 nm (f_{PSII_760}) and 740 nm (f_{PSII_740}). The colored shapes and error bars denote the mean and standard deviation for 52 species, respectively, while the gray dots show the results for each species.

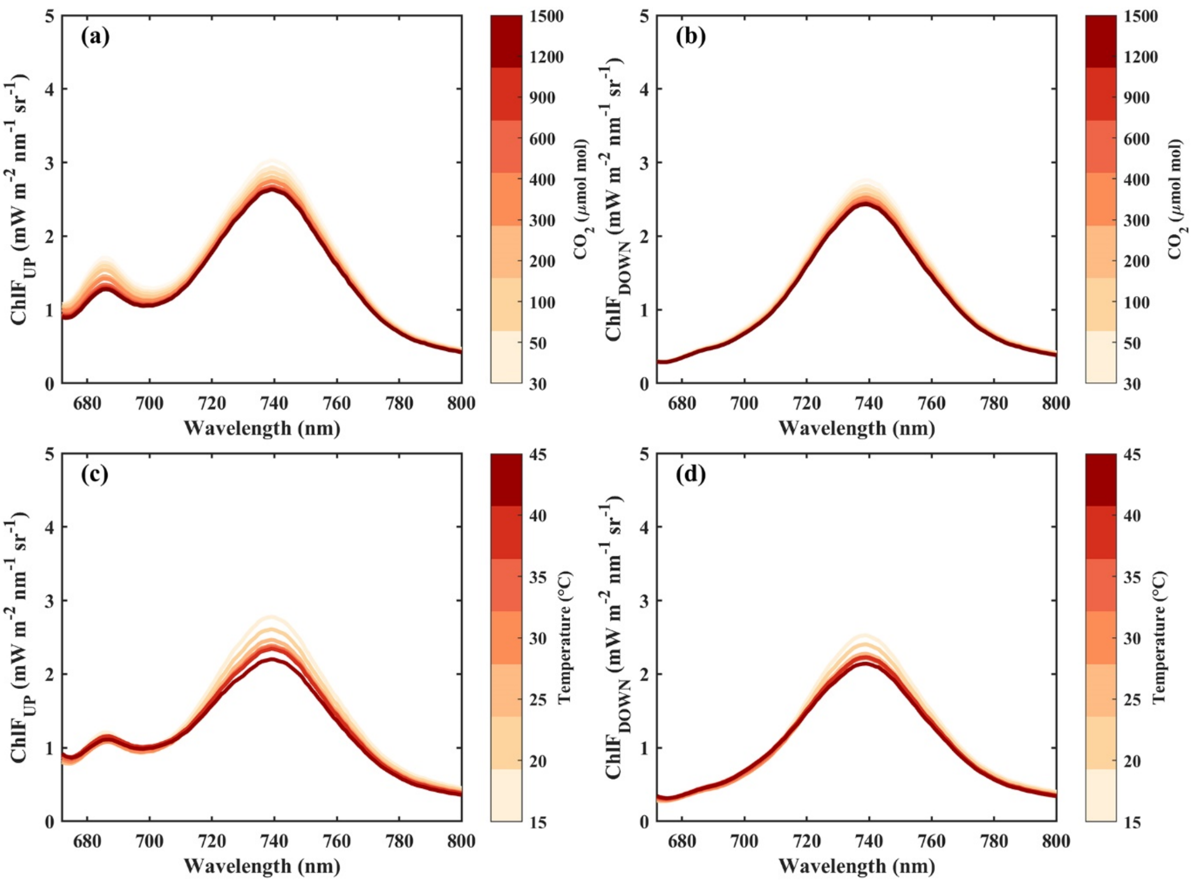

Figure S3. CO_2 response curve of (a) upward chlorophyll fluorescence ($ChlF_{UP}$, mW m^{-2} nm^{-1} sr^{-1}) and (b) downward chlorophyll fluorescence ($ChlF_{DOWN}$, mW m^{-2} nm^{-1} sr^{-1}). Temperature response curve of (c) upward chlorophyll fluorescence ($ChlF_{UP}$, mW m^{-2} nm^{-1} sr^{-1}) and (d) downward chlorophyll fluorescence ($ChlF_{DOWN}$, mW m^{-2} nm^{-1} sr^{-1}).

Table S1. The fitted parameters (m_{opt}, H_d, H_a, and T_{opt}) of the ChlF$_{PSII}$-q_L relationship for different species.

PFTs	Species	m_{opt}	H_d (J mol^{-1})	H_a (J mol^{-1})	T_{opt}
ENF	Pinus massoniana Lamb.	2.04	131600	18309	40.76
	Pinus bungeana Zucc.	2.40	179400	28451	35.80
	Abies fabri (Mast.) Craib	2.13	432500	17751	39.85
	Cedrus deodara (Roxb.) G. Don	2.0	112300	21166	31.89
	Pinus thunbergii Parl.	3.39	549900	23308	42.04
	Cunninghamia lanceolata (Lamb.) Hook.	1.38	138800	27775	36.81
	Pinus sylvestris var. mongholica Litv.	2.50	2000700	19326	43.78
	Mean ± std	2.26 ± 0.62	506450 ± 680450	22298 ± 4389	38.71 ± 4.10
EBF	Chrysanthemum × morifolium Ramat	2.43	248110	18979	38.01
	Eriobotrya japonica (Thunb.) Lindl.	2.94	355200	211225	40.13
	Trachycarpus fortunei (Hook.) H. Wendl.	1.95	63600	48755	36.56
	Osmanthus fragrans (Thunb.) Lour.	3.35	387300	29049	42.00
	Magnolia grandiflora L	1.51	160710	19170	34.54
	Schefflera heptaphylla (Linnaeus) Frodin	1.40	183470	14879	36.35
	Mean ± std	2.26 ± 0.79	233070 ± 122760	25343 ± 12385	37.94 ± 2.73
DNF	Metasequoia glyptostroboides Hu & W. C. Cheng	1.49	225200	12645	38.22
	Pinus parviflora Siebold et Zuccarini	1.31	147410	65952	37.33
	Pseudolarix amabilis (Nelson) Rehd.	1.60	180300	26263	34.05
	Mean ± std	1.45 ± 0.15	18430 ± 39048	34953 ± 27696	36.53 ± 2.19
DBF	Aesculus chinensis Bunge	2.74	329720	20180	41.46
	Aesculus chinensis Bunge	1.33	166710	9300	35.82
	Robinia pseudoacacia L.	2.12	158240	19960	35.95
	Prunus persica L.	3.00	160180	26280	41.25
	Wisteria sinensis (Sims)Sweet	1.85	176420	20210	35.00
	Ginkgo biloba L.	2.41	197990	19110	34.31
	Ginkgo biloba L.	1.60	157000	19810	37.11
	Xanthoceras sorbifolium Bunge	1.38	163100	15760	36.38
	Morus alba cv.Pendula	1.76	395560	16880	39.20
	Fraxinus mandshurica Rupr.	1.52	137110	15090	33.26
	Robinia pseudoacacia cv. idaho	0.94	576920	566570	23.82
	Mean ± std	1.88 ± 0.63	238090 ± 139050	68014 ± 165410	35.78 ± 4.78

SHR	Paeonia × suffruticosa Andrews	3.03	183400	25602	40.48
	Rosa chinensis Jacq.	2.43	145550	26566	33.70
	Viburnum melanocarpum Hsu	1.67	92740	28047	31.84
	Acer negundo L.	2.48	180940	21477	37.25
	Armeniaca mume Sieb.	3.07	321230	21449	41.27
	Ilex cornuta Lindl. et Paxt.	1.63	111330	16557	36.11
	Periploca sepium Bunge	1.64	275270	15791	41.11
	Photinia serratifolia (Desfontaines) Kalkman	2.90	349730	13684	40.42
	Cornus alba Linnaeus	3.12	444410	20841	40.53
	Mean ± std	2.44 ± 0.64	233850 ± 119990	21113 ± 5032	38.08 ± 3.52
GRA	Ligustrum lucidum Ait.	1.74	179180	22094	37.19
	Lactuca seriola L	2.76	502940	22241	41.98
	Ficus tikoua Bur.	3.05	227200	20020	37.65
	Solanum lyratum Thunb.	1.13	232900	20881	38.48
	Setaria viridis (L.) Beauv.	1.26	170450	12076	37.07
	Phytolacca americana L.	2.05	155610	17523	39.33
	Cayratia japonica (Thunb.) Gagnep.	3.20	196020	24673	40.78
	Iris tectorum Maxim.	2.87	152900	24998	41.50
	Canna indica L.	2.59	60090	34541	35.22
	Ophiopogon bodinieri Levl.	1.49	190410	14257	37.71
	Panicum virgatum L.	3.85	357690	35589	40.89
	Achyranthes bidentata Blume	1.65	175610	18112	36.23
	Mean ± std	2.30 ± 0.87	216750 ± 113160	22250 ± 7111	38.67 ± 2.20
CRO	Beta vulgaris L.	2.50	145000	22669	40.53
	Capsicum annuum L.	1.77	87600	28494	38.01
	Panicum italicum L.	2.53	5521800	22429	44.67
	Sorghum bicolor (L.) Moench	2.95	185400	26321	37.96
	Mean ± std	2.45 ± 0.49	1484900 ± 2691500	24978 ± 2943	40.29 ± 3.15

References

1. Liu, Zhunqiao, Feng Zhao, Xinjie Liu, Qiang Yu, Yunfei Wang, Xiongbiao Peng, Huanjie Cai, and Xiaoliang Lu. "Direct Estimation of Photosynthetic Co2 Assimilation from Solar-Induced Chlorophyll Fluorescence (Sif)." *Remote Sensing of Environment* 271 (2022): 112893.
2. Palombi, Lorenzo, Giovanna Cecchi, David Lognoli, Valentina Raimondi, Guido Toci, and Giovanni Agati. "A Retrieval Algorithm to Evaluate the Photosystem I and Photosystem Ii Spectral Contributions to Leaf Chlorophyll Fluorescence at Physiological Temperatures." *Photosynthesis Research* 108 (2011): 225-39.
3. Magney, Troy S, Christian Frankenberg, Philipp Köhler, Gretchen North, Thomas S Davis, Christian Dold, Debsunder Dutta, Joshua B Fisher, Katja Grossmann, and Alexis Harrington. "Disentangling Changes in the Spectral Shape of Chlorophyll Fluorescence: Implications for Remote Sensing of Photosynthesis." *Journal of geophysical research: Biogeosciences* 124, no. 6 (2019): 1491-507.
4. Van der Tol, C, W Verhoef, J Timmermans, Anne Verhoef, and Zhongbo Su. "An Integrated Model of Soil-Canopy Spectral Radiances, Photosynthesis, Fluorescence, Temperature and Energy Balance." *Biogeosciences* 6, no. 12 (2009): 3109-29.
5. Van der Tol, C, JA Berry, PKE Campbell, and U Rascher. "Models of Fluorescence and Photosynthesis for Interpreting Measurements of Solar ‐ Induced Chlorophyll Fluorescence." *Journal of geophysical research: Biogeosciences* 119, no. 12 (2014): 2312-27.
